# Online Opioid Stewardship Training for Long-Term Care Staff

**DOI:** 10.1093/geroni/igaa057.1721

**Published:** 2020-12-16

**Authors:** Linda Edelman, Troy Andersen, Cherie Brunker, Nicholas Cox, Jorie Butler, Miguel Galindo, Larry Garrett, Susan Butterwork

**Affiliations:** 1 University of Utah, Salt Lake City, Utah, United States; 2 University of Utah - College of Pharmacy, Salt Lake City, Utah, United States; 3 University of Utah - School of Medicine, Salt Lake City, Utah, United States; 4 University of Utah - College of Nursing, Salt Lake City, Utah, United States; 5 Q-consult, LLC, St. Petersburg, Florida, United States

## Abstract

Opioids are often the first-line chronic pain management strategy for long-term care (LTC) residents who are also at increased risk for opioid-related adverse events. Therefore, there is a need to train LTC providers and staff about appropriate opioid use and alternative treatment strategies. Our interdisciplinary team worked with LTC partners to identify staff educational needs around opioid stewardship. Based on this need’s assessment, we developed eight modules about opioid use and risks for older adults, including those with dementia, recommendations for de-prescribing including other pharmacological and non-pharmacological alternatives, SBIRT, and motivational interviewing to determine “what matters”. Each 20-minute module contains didactic and video content that is appropriate for group staff training or individuals and provides rural LTC facilities access to needed training in their home communities. Within the first month of launching online, the program received over 1100 hits and LTC partners are incorporating modules into clinical staff training schedules.

